# Developing Equations for Free Vibration Parameters of Bistable Composite Plates Using Multi-Objective Genetic Programming

**DOI:** 10.3390/polym14081559

**Published:** 2022-04-11

**Authors:** Saeid Saberi, Alireza Sadat Hosseini, Fatemeh Yazdanifar, Saullo G. P. Castro

**Affiliations:** 1 Department of Mechanical Engineering, Isfahan University of Technology, Isfahan 84156-83111, Iran; s.saberi@alumni.iut.ac.ir; 2 School of Civil Engineering, College of Engineering, University of Tehran, Tehran 14179-35840, Iran; a.sadat@ut.ac.ir; 3 Department of Mathematics and Computer Science, Amirkabir University of Technology, Tehran 15875-4413, Iran; fatemehyazdanifar400@gmail.com; 4 Department of Aerospace Structures and Materials, Delft University of Technology, 2629 HS Delft, The Netherlands

**Keywords:** bistable, composite, vibration, FEM, genetic programming, sensitivity

## Abstract

For the last three decades, bistable composite laminates have gained publicity because of their outstanding features, including having two stable shapes and the ability to change these states. A common challenge regarding the analysis of these structures is the high computational cost of existing analytical methods to estimate their natural frequencies. In the current paper, a new methodology combining the Finite Element Method (FEM) and Multi-Objective Genetic Programming (MOGP) is proposed for the analysis of bistable composite structures, leading to some analytical relations derived to obtain the modal parameters of the shells. To achieve this aim, the data extracted from FEM, consisting of the ratio of the length to width (*a*/*b*) and the thickness (*t*) of the laminate, is split into Train and Validation, and Test, subsets. The former is used in MOGP, and four formulas are proposed for the prediction of the free vibration parameters of bistable laminates. The formulas are checked against the Test subset, and the statistical indices are calculated. An excellent performance is observed for all GP formulas, which indicates the reliability and accuracy of the predictions of these models. Parametric studies and sensitivity analyses are conducted to interpret the trend of input parameters in the GP models and the level of sensitivity of each natural frequency formula to the input parameters. These explicit mathematical expressions can be extended to the other bistable laminates to obtain their natural frequencies on the basis of their geometrical dimensions. The results are validated against the experimental data and verified against FEM outcomes.

## 1. Introduction

Bistable composite laminates can be designed to render two stable static configurations that are suitable candidates for deployable and aero structures, including booms [1], airfoils [2], winglet and wing [3], and trailing edge [4], as well as energy-harvesting devices [5,6,7] and other morphing structures [8,9,10,11,12].

**Figure 1:**
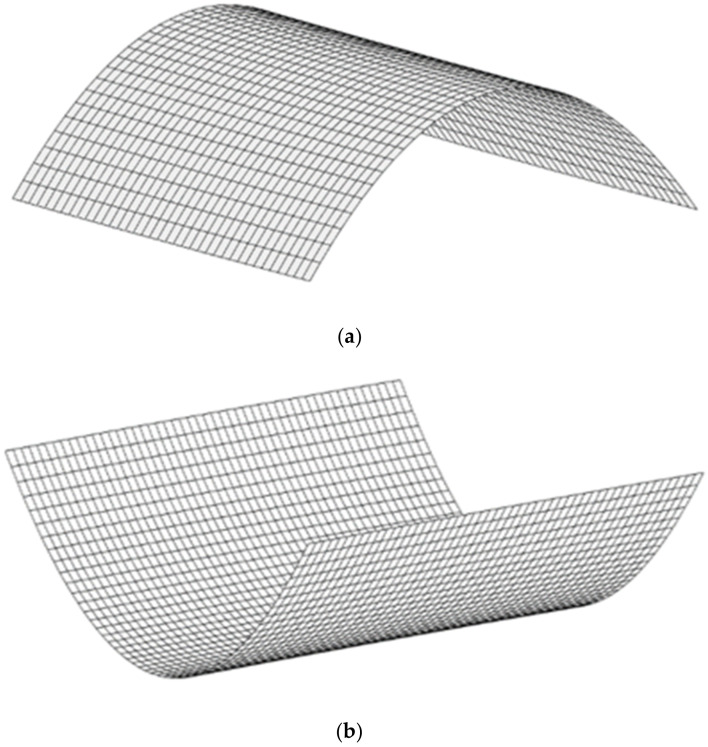
Two stable equilibria shapes of a cross-ply bistable composite plate, (**a**) the first stable (**b**) the second stable configuration.

In 1981, Hyer [13] found that an unsymmetric composite laminate could have two cylindrical stable shapes besides its statically unstable saddle shape. Indeed, after the manufacturing process, a cross-ply bistable laminate exhibits two cylindrical stable positions, as illustrated in Figure 1.

Due to the fact that the transverse and longitudinal thermal expansion coefficients of the laminate mismatch, the thermal residual stresses are created during the fabrication process, resulting in the making of a nonlinear geometry feature and the emergence of two stable shapes [14]. Hyer proposed an analytical model, based on the principle of minimum energy and the Rayleigh–Ritz method, by taking into account this geometrical nonlinearity [15,16]. Ren and Majidi [17] obtained the cured shape of different cross-ply composite shells, investigating the effect of the thickness, size, and stacking sequence on the predicted shapes through utilizing the suggested method by Hyer [13]. Mattioni et al. [18] presented a methodology to provide a numerical prediction of the nonlinear responses of bistable and multistable laminates under cool-down and snap-through conditions utilizing finite element analyses. Betts et al. [19] investigated the stable configurations of angle-ply bistable plates using an analytical method, and the results were justified comparing the surface profiles obtained by experimental tests. According to Saberi et al. [20], besides the stacking sequences, the material properties, geometrical features, and environmental conditions have a noticeable impact on the nonlinear behaviors of bistable structures. Saberi et al. [21] estimated the probability of five types of bistable composite laminates using the principle of minimum energy, applying the Rayleigh–Ritz method with the subset simulation technique, which is an efficient approach to predict small failure probabilities. They considered the material properties, environmental conditions, and geometry characterizations as input parameters, treated simultaneously to determine the sensitivity of the bistable region. The reliability-based sensitivity analysis showed that for bistable laminates subject to humidity, the coefficient of thermal and moisture expansions has the greatest impact on the bistability probability. However, for bistable laminates without moisture, the thickness became the most important factor. Brampton et al. [22] studied the influence of geometrical uncertainties, environmental conditions, and material properties on the major curvature of bistable composite shell. The sensitivity of the cured shape to each variable was obtained by considering a ±5% change in the variable using the Rayleigh–Ritz method. Chai et al. [23] studied the impact of moisture and temperature on the bistable antisymmetric composite shell, presenting a new model to control the curvatures of a bistable shell by adjusting the temperature or moisture. Fazli et al. [24] investigated the bistability of the hexagonal laminates and the compound symmetric trapezoidal with two varied lay ups, studying the influences of the size of the lamina and stacking sequence on the out-of-plane displacement. Deshpande et al. [25] examined, numerically and experimentally, the transient process between two stable shapes of a bistable laminate during snap-through and approved that transient deformation depends on the location of the applied force. Phatak et al. [26] defined a relationship between the behavioral and geometrical dimensions of bistable laminates, which can predict their curvature and snap-through force. It was shown that the force is a function of the thickness and the side length. Nicassio [27] presented a novel analytical method based on Timoshenko and Ashwell’s theories in order to obtain the stable configurations of bistable plates. He verified the results with experimental and FE methods results. Pietro et al. [28] introduced a computational framework based on advanced one-dimensional finite elements to analyze bistable beam structures and predict the in-plane stress field with acceptable accuracy. Kuang et al. [29] analyzed the multi-stability of composite shells using a new numerical framework that can compute the nonlinear equilibrium paths and assess the stability of the equilibrium structure. Zhang et al. [30] designed the multistable composite structures, made by connecting some bistable laminates, and obtained their stable configurations.

Most of the papers about the bistable composite laminates focus on the static behavior, and few authors have investigated the vibration and dynamic responses. Diaconu et al. [31] studied the dynamic transition between stable states for a bistable composite plate through Hamilton’s principle and the Rayleigh–Ritz method. Arrieta et al. [32] experimentally and theoretically investigated the cross-well dynamic response of a bistable composite laminate. Wu and Viquerat [33] optimized the natural frequency of the bistable composite slit tubes with cantilevered conditions with respect to the size and stacking sequences using Matlab’s standard optimization functions and finite elements. Wu et al. [34] presented a novel analytical model founded on Hamilton’s principle and Riley Ritz methods to predict the nonlinear dynamic behavior of the bistable composite laminate. Emam [35] studied the free vibration response of cross-ply bistable composite laminates through a simplified Rayleigh–Ritz model as well as the effect of geometrical size of laminate on the fundamental natural frequency, whose results indicated the natural frequency declines if the laminate length rises. Wu et al. [36] investigated the nonlinear vibration behaviors of the cross-ply bistable composite shallow shell under centre foundation excitation by finite elements and experimental methods. They found that the desired shell varies from the single-well periodic to single-well chaotic vibrations through the period-doubling bifurcation because of the growth of the amplitudes for the centre base excitations. Andrew and Inman [37] developed a model to study the nonlinear vibration response of a bistable laminate combined with a central macro fibre composite and studied the full range of nonlinear behaviors such as chaotic, limit cycle, and subharmonic oscillations. Brunetti et al. [38] investigated, experimentally and theoretically, the nonlinear dynamic response of a cantilever bistable shell. Liu et al. [39] observed the metastable chaos of a square bistable shell under foundation excitation and found that the zero equilibrium point is unstable once the parametric excitation increases from a specific critical value. Dong et al. [40] studied the global and local dynamic response of a bistable asymmetric laminated shell under the base excitation by changing temperature, detuning parameters, and base excitation amplitude. They showed that for large amplitude, the shell vibrates between the two stable configurations, which is dominated by the global dynamics. Most of the aforementioned papers investigated the dynamic response and obtained just the first natural frequency of bistable laminates since determining other natural frequencies using the analytical method is challenging. Recently, Saberi et al. [41] developed an analytical equation using deep learning and the FEM to estimate the modal parameters of rectangular bistable composite plates.

The current study presents new mathematical expressions using Multi-Objective Genetic Programming (MOGP) to predict the first four natural frequencies of bistable composite laminates through their geometrical sizes. To determine these analytical terms, at the first step, the appropriate signals are collected from the vibration of the laminate through FEM as well as using the experimental results. Afterwards, the data are divided into two separate categories: the train and validation group; and the test group. The former is employed by the genetic programming (GP) algorithm in a recursive process, whereas the latter is utilized to check the performance of the predictions. Four formulas are obtained for each natural frequency of the bistable laminates based on their geometrical parameters. The high accuracy of the formulae is verified after examining the correlation of coefficients and mean relative error of the models for both datasets. The obtained expressions help to state the output (natural frequencies) based on the inputs (length, width, and thickness of laminate) using explicit nonlinear mathematical relationships. In addition, the sensitivity of the natural frequencies with respect to these parameters is investigated.

## 2. Theoretical Formulation of a Bistable Laminate

To estimate the stable positions, the strain terms of Von Kármán should be considered. The total number of strains is expressed in Equation (1)

(1) $$\left\{\begin{array}{c} \varepsilon_{xx}\\ \varepsilon_{yy}\\ \varepsilon_{xy} \end{array}\right\}=\left\{\begin{array}{c} \varepsilon^{0}{}_{xx}\\ \varepsilon^{0}{}_{yy}\\ \varepsilon^{0}{}_{xy} \end{array}\right\}+z\left\{\begin{array}{c} \kappa^{0}{}_{xx}\\ \kappa^{0}{}_{yy}\\ \kappa^{0}{}_{xy} \end{array}\right\}$$

where $\left(i,j=x,y\right)\;\varepsilon^{0}{}_{ij}$ is the strain vector in the mid-plane and $\kappa^{0}{}_{ij}$ is the curvature in the mid-plane, which are defined according to Equation (2).

(2) $$\left[\begin{array}{c} \varepsilon^{0}{}_{xx}\\ \varepsilon^{0}{}_{yy}\\ \varepsilon^{0}{}_{xy} \end{array}\right]=\left[\begin{array}{c} \frac{\partial U_{0}}{\partial x}+\frac{1}{2}{\left(\frac{\partial W_{0}}{\partial x}\right)}^{2}\\ \frac{\partial V_{0}}{\partial y}+\frac{1}{2}{\left(\frac{\partial W_{0}}{\partial y}\right)}^{2}\\ \frac{1}{2}\left(\frac{\partial U_{0}}{\partial y}+\frac{\partial V_{0}}{\partial x}+\frac{\partial W_{0}}{\partial x}\frac{\partial W_{0}}{\partial y}\right) \end{array}\right]\;\mathrm{and}\;\left[\begin{array}{c} \kappa^{0}{}_{xx}\\ \kappa^{0}{}_{yy}\\ \kappa^{0}{}_{xy} \end{array}\right]=\left[\begin{array}{c} -\frac{\partial^{2}W_{0}}{\partial x^{2}}\\ -\frac{\partial^{2}W_{0}}{\partial y^{2}}\\ -2\frac{\partial^{2}W_{0}}{\partial x\partial y} \end{array}\right]$$

In Equations (1) and (2),$\;U_{0},\;V_{0},\;\mathrm{and}\;W_{0}\;$ indicate the in-plane and out-of-plane displacements in the *x*, *y,* and *z* and directions, at the mid-plane of plate. The displacement fields are considered as:

(3) $$U_{0}{}^{\;}\left(x,y\right)=\overset{m}{\underset{i=1}{\sum }}\overset{n}{\underset{j=1}{\sum }}a_{ij}x^{i}y^{2\left(j-1\right)}\;V_{0}{}^{\;}\left(x,y\right)=\overset{m}{\underset{i=1}{\sum }}\overset{n}{\underset{j=1}{\sum }}b_{ij}x^{2\left(i-1\right)}y^{j}\;W_{0}\left(x,y\right)=\overset{m}{\underset{i=1}{\sum }}\overset{n}{\underset{j=1}{\sum }}c_{ij}x^{2\left(i-1\right)}y^{2\left(j-1\right)}$$

where $a_{ij}\left(t\right),$ $b_{ij}\left(t\right),$ and $c\left(t\right)$ are unknown coefficients that illustrate the generalized coordinates, and their quantity is equal to 3×m×n. $R\left(t\right)={\left\{a_{ij},b_{ij},c_{ij}\right\}}^{T}\;$ is the vector of the unknown coefficients. It should be mentioned that *m* and *n* are the degrees of freedom. These forms can satisfy the boundary conditions of the laminate, which is supposed to fix in the middle point and be free at the edges. Regarding the Kirchhoff hypothesis, the displacement in each point of plate is defined by Equation (4).

$$U\left(x,y,z,t\right)=U_{0}{}^{\;}\left(x,y,t\right)-z\frac{\partial W_{0}\left(x,y,t\right)}{\partial x}\;V\left(x,y,z,t\right)=V_{0}{}^{\;}\left(x,y,t\right)-z\frac{\partial W_{0}\left(x,y,t\right)}{\partial y}$$

(4) $$W\left(x,y,z,t\right)=W_{0}{}^{\;}\left(x,y,t\right)$$

Hamilton’s principle is employed to derive the desired dynamic equations of the bistable laminate. The stationary action functional is obtained by integrating the Lagrangian functional between two time instants ($t_{1}$ and $t_{2})$. Hamilton’s principle states that a dynamic system seeks motion within a path where the action functional is stationary. This stationary point is given by:

(5) $$\delta \overset{t_{2}}{\underset{t_{1}}{\int }}\left(T\left(t\right)-\Pi \left(t\right)\right)dt=0$$

where $\Pi \left(t\right)$ is the total potential energy and $T\left(t\right)$ is the kinetic energy.

The total potential energy of a composite laminate, ignoring any mid-plane offset [42,43], is described in Equation (6).

(6) $$\Pi =\overset{L_{y}/2}{\underset{-L_{y}/2}{\int }}\overset{L_{x}/2}{\underset{-L_{x}/2}{\int }}(\frac{1}{2}{\left[\begin{array}{c} \varepsilon^{0}\\ \kappa^{0} \end{array}\right]}^{T}\left[\begin{array}{cc} A & B\\ B & D \end{array}\right]\left[\begin{array}{c} \varepsilon^{0}\\ \kappa^{0} \end{array}\right]-{\left[\begin{array}{c} N^{t}\\ M^{t} \end{array}\right]}^{T}\left[\begin{array}{c} \varepsilon^{0}\\ \kappa^{0} \end{array}\right])dxdy$$

where $\;L_{x}$ and $L_{y}$ are the dimensions of the the plate and *A*, *B,* and *D* are, respectively, the in-plane, coupling, and bending stiffness matrices. $N^{t}$ and $M^{t}$ are the thermal stress and moment resultants, respectively. The kinetic energy of a composite laminate, ignoring again any mid-plane offset, is defined as:

(7) Tt=12∫−Ly2 Ly2 ∫−Lx2Lx2 ∫−h2h2 ρ∂U∂t2+∂V∂t2+∂W∂t2dzdxdy

where $L_{x}$, $L_{y}$, and *h* are the lengths and thickness of the laminate and ρ is the density.

Then, by substituting the Equations (6) and (7) into Equation (5) and integrating, the dynamic equations of motion are stated as:

(8) $$M\ddot{R}+D\left(\dot{R}\right)+K\left(R\right)=0$$

where M is the mass matrix that is obtained by integrating and $\;D\left(\dot{R}\right)$ is the damping matrix expressed as:

(9) $$D\left(\dot{R}\right)=\alpha M\dot{R}$$

where α is the coefficient of the mass proportional damping [41].

$K\left(R\right)$ is the nonlinear stiffness function that is determined by Equation (10).

(10) $$K\left(R\right)=\frac{\partial \Pi }{\partial R_{\;}}=\left[\begin{array}{ccc} \frac{\partial^{2}\Pi }{\partial a_{ij}{}^{2}} & \frac{\partial^{2}\Pi }{\partial a_{ij}\partial b_{ij}} & \frac{\partial^{2}\Pi }{\partial a_{ij}\partial c_{ij}}\\ \frac{\partial^{2}\Pi }{\partial b_{ij}\partial a_{ij}} & \frac{\partial^{2}\Pi }{\partial b_{ij}{}^{2}} & \frac{\partial^{2}\Pi }{\partial b_{ij}\partial c_{ij}}\\ \frac{\partial^{2}\Pi }{\partial c_{ij}\partial a_{ij}} & \frac{\partial^{2}\Pi }{\partial c_{ij}\partial b_{ij}} & \frac{\partial^{2}\Pi }{\partial c_{ij}{}^{2}} \end{array}\right]$$

The equilibrium configuration of the plate will stable if this Jacobian matrix (*K* (*R*)) of the potential energy is positive definite.

## 3. Numerical and Experimental Methods

A parametric finite element is used to extract the natural frequencies for different geometries and laminate configurations. The resulting data are then used as the input of the GP algorithm. In order to perform the finite element analyses, the ABAQUS software package is employed. The studied bistable composite plate has a stacking sequence [0/90] and 200 mm × 140 mm side length. The material properties of this laminate are presented in Table 1.

**Figure 2:**
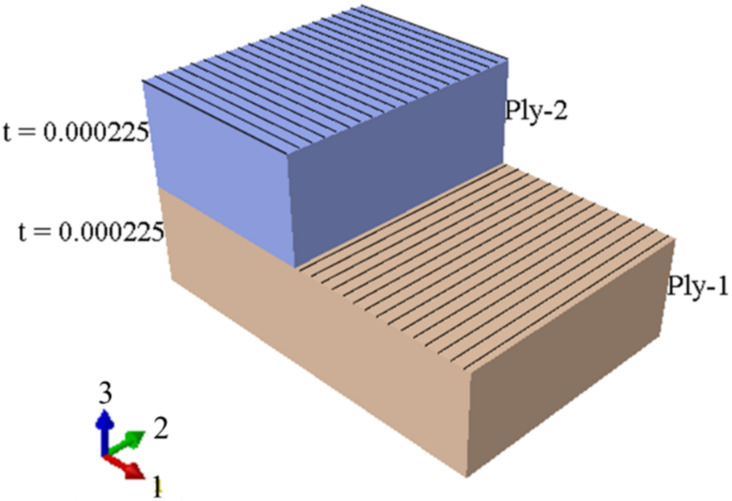
The schematic of the simulated laminate, comprising two plies.

After designing the desired plate and applying the material properties, the simulated laminate is depicted in Figure 2.

The simulated laminate is discretized using linear quadrilateral elements with reduced integration and hourglass control (S4R), and the converged mesh consists of 1189 nodes and 1120 elements to represent the plate geometry. To determine the stable configurations, the “Static-General” step should be selected since simulating the curing process is possible in this step. Meanwhile, because of the geometric nonlinearity, “Nlgeom” is chosen for the static analysis. What is more, to reach reliable and robust results, it is needed to use a damping factor of 10^−7^ for automatic stabilization [31,44,45].

To simulate the curing process, the temperature variation should be defined using the “Predefined Field” so that at the initial step the temperature is 180 °C and for other steps is equal to room temperature (20 °C).

**Figure 3:**
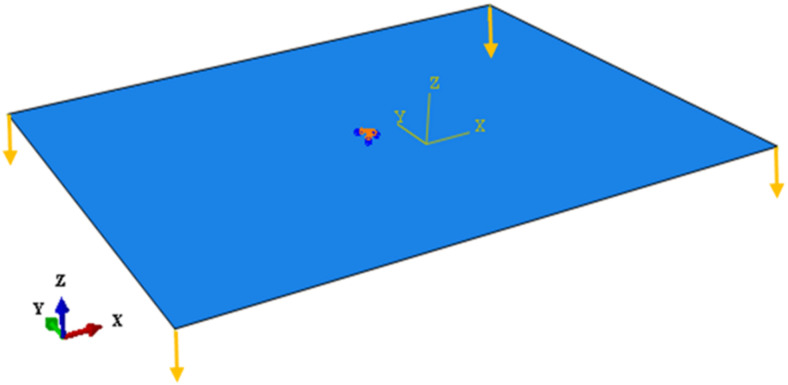
The laminate with the clamped center, and four vertical forces in the corners.

Since the studied bistable plate is rectangular, the determination of the first stable shape does not need the primary imperfection but should be applied to obtain the second stable state, in order to apply the conditions of the manufacturing process. To achieve this aim, two “Static-General” steps are defined, and then the imperfection is introduced in the first step by applying 4-min vertical forces (for this case, each force is about 0.02 N) to the corners, removed in the second step (regarding Figure 3). Indeed, these initial forces simulate imperfections in materials and manufacturing conditions through manufacturing process.

**Figure 4:**
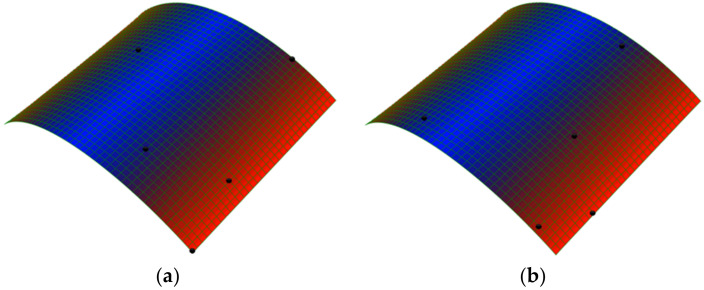
The random points chosen to apply load (**a**) and to extract the dynamic response of the bistable plate (**b**).

After modelling the stable shapes, the vibrational response in these states ought to extract. First, the “Dynamic Implicit” step is selected. To simulate the free vibration behaviour in this step, a very small force, as the initial actuation, is initially applied to the plate, resulting in a transverse displacement. Then, the small force is removed such that the proper transverse displacement response of the laminate can extracted from some random points defined on the laminate. To determine a dynamic response with high accuracy, the time step in this step should be small. The points used to apply the forces and receive response are shown in Figure 4.

**Figure 5:**
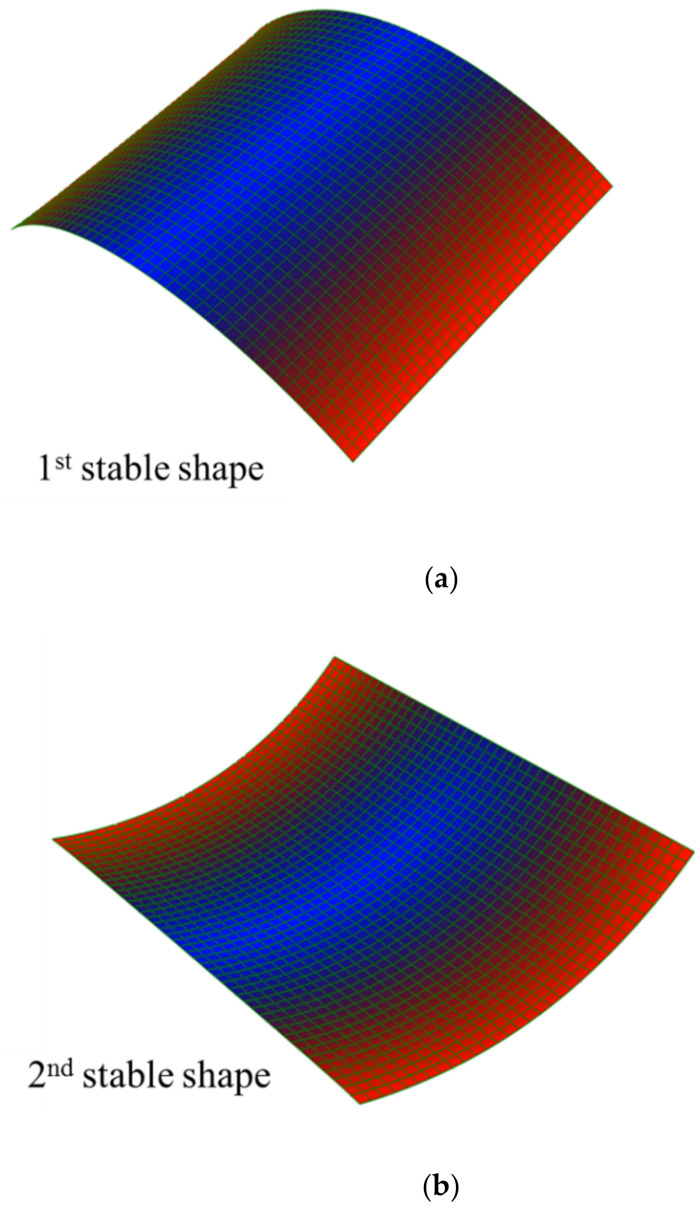
Stable shapes of a cross-ply (0/90) bistable composite, (**a**) the first stable (**b**) the second stable shape.

In order to obtain the natural frequencies of the plate in each stable state, after simulating the static stable configurations, the “Frequency” step is considered for investigating the free vibration parameters by using the “Lanczos” eignsolver. The sable configurations of a cross-ply [0/90] bistable composite are illustrated in Figure 5. In the case of boundary conditions, during “Static-General” steps the middle point of the plate is clamped (‘’Encaster’’ boundary condition) (according Figure 3), but for the ‘’Frequency’’ step, in order to remove rotational rigid body modes, the three translational degrees-of-freedom are constrained (‘’Pinned’’ boundary condition).

**Figure 6:**
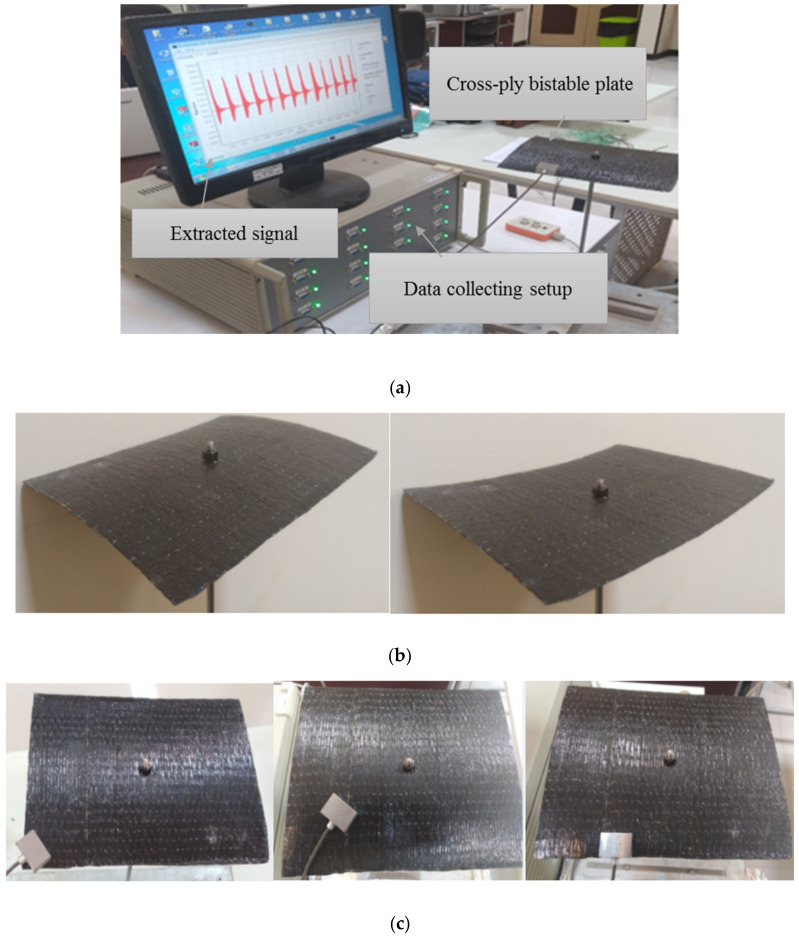
Experimental method to collect the desired signal from the vibrational response of a bistable composite plate: (**a**) the used set up, (**b**) the two stable shapes of the bistable laminate, and (**c**) an appropriate sensor in different points of the laminate.

For the experimental identification of the natural frequencies, at first, the centre of the plate is fixed via a steel rod that is joined to the plate using a bolt that provides a point clamp at the centre. A proper sensor is attached in varied points of two stable configurations of laminate, and this accelerometer is linked to the data acquisition hardware that is connecting to a computer. Then, to simulate the primary force, a low actuation is applied to some random points of the laminate using a plastic impact hammer. Lastly, the signals associated with the transverse displacement of the plate are extracted by a special set-up for the modal test. The experimental test set-up is depicted in Figure 6.

## 4. Genetic Programming Methodology

Genetic programming (GP), first introduced by Koza [46], is a powerful algorithm for predicting meaningful relationships between input and output variables [47,48,49,50]. GP follows the Darwinian principle of survival and reproduction in a population and in genetic crossover operations. For a problem including constant quantities and variables as terminals, a random composition of the algebraic and logical functions as the population of the first generation is prepared. A new offspring of the initial generation is generated through mutation and crossover, also known as recombination, based on the genetic information of the parents. The recombination results in the increase of the variation of population, generating new individuals. Fitness values are checked for every generation, and those who have the highest fitness values have more chance of remaining in the next-generation population. This process is in the form of a tree in which the root node is the starting point, nodes of the tree are the genes containing functions and/or terminals, and branches are the connectors of these functions and terminals. A new language was developed by Karma [51] for reading the chromosomes’ information, consisting of letters used to represent the variables and constants in the form of K-expressions in a computer program. A multi-gene model consists of some genes that can be presented in the form of distinctive trees, where each one corresponds to a symbolic mathematical expression. A weighted linear combination links these expressions and generates a formula as:

(11) $$\hat{y}\left(x,w,r\right)=w_{0}+\overset{n}{\underset{i=1}{\sum }}w_{i}G_{i}\left(r,x\right)$$

where *n* stands for the number of genes, and *x* and *r* are the input and the vector of unknown parameters in each gene, respectively, in a vector of outputs, *G*. *w_i_* is the weight of *i*th gene and *w_0_* is the bias term. What makes MOGP a reliable tool for developing formulations for a set of data is its relatively higher efficiency and capability in modelling nonlinear complex problems [52]. MOGP can optimize the complexity development and the goodness of fit to restrict the model from over-expansion and over complexity.

In this study, the output dataset from the FE analysis is introduced to the MOGP toolbox [53] and the initial population. In this algorithm, in every generation of the population, the top 50% of the population regarding the accuracy and complexity survive to the next population. More information on the details of this algorithm can be found in Searson [53].

The inputs of the model comprise two parameters, the *a*/*b* length-to-width geometric ratio and the thickness *t*. The output parameters are the first four natural frequencies of the laminate. These parameters are introduced to the MOGP algorithm, and through running evolutionary algorithms on the generated populations as described before, an appropriate and optimum set of model parameters for the present problem is found. Table 2 illustrates the model parameters of the MOGP models. The complexity of the models is determined by the maximum number of genes and the depth of the trees.

## 5. Results

### 5.1. Determining the Natural Frequencies

To achieve the natural frequency using the theoretical method, Equation (8) can be solved through Runge–Kutta method, in which the results will be the time response of the laminate (*R* vector). To extract this dynamic response, first, four equal concentrated forces of 1.10 *N* are initially applied at each corner of the plate, and then these forces are eliminated. It should be noticed that in order to capture the linear vibration behaviour of the bistable laminate without jumping into the other stable shape, the applied corner forces must stay below the levels corresponding to the snap-through. In the experimental method, the natural frequency is obtained from the peak of the frequency response function that is calculated from the cross-power spectrum density between the frequency-domain response of the accelerometer and impact hammer, and the power spectrum density of the frequency-domain response of the impact hammer. These frequency-domain responses are obtained using a Fast Fourier Transform (FFT) applied to the time-domain response of the impact hammer and accelerometer. Regarding the bistable composite plate with the properties proposed in Table 1, the natural frequency determined by the analytical method is equal to 35.47 Hz, which is in good agreement with the natural frequency determined experimentally that is 34.56 Hz.

**Figure 7:**
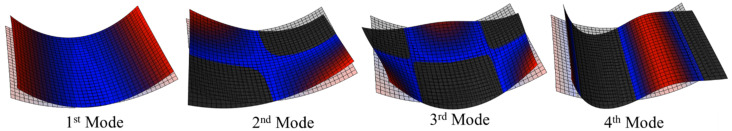
Associated mode shapes with the first four natural frequencies around the first stable configuration.

Table 3 and Figure 7 present the first four natural frequencies and their associated modes predicted with the finite element model, respectively.

To increase the accuracy of the results, we should consider more terms (high-order terms) in Equation (3), leading to the substantial enhancement of the computational cost. In addition, solving Equation (8) to receive a dynamic response is time-consuming because to gain acceptable precision, the time step should be sufficiently small. Therefore, four formulas are offered to estimate these parameters with acceptable accuracy and without any complexity.

### 5.2. Mathematical Expressions

Two input parameters, including *x*_1_ = *a*/*b* and *x*_2_ = *t*, contribute to the generation of the GP population and training-validation process. Several runs are conducted, and according to the explanations of Section 4, four GP models with the highest performance are achieved for the natural frequencies of the composite laminates that are presented in Table 4.

The GP models need to be evaluated using well-known criteria such as the Frank and Todeschini [54] model acceptability criterion. The ratio of the number of data points to the number of input parameters should be greater than five. For the GP models of this study, this ratio is 157 and fulfils the acceptability of the model. Another criterion by Smith [55] says that the error value (the Mean Relative Error in this study) must be at a minimum value regarding the acceptable errors of prediction in this problem, i.e., less than 2.15 percent, while the correlation of coefficients has to be higher than 0.8. The formulation of R^2^ and *MRE* are as follows:

(12) $$\hat{y}\left(x,w,r\right)=w_{0}+\overset{n}{\underset{i=1}{\sum }}w_{i}G_{i}\left(r,x\right)$$

(13) $$MRE=\frac{1}{n}\sum \left|\frac{F-G}{F}\right|$$

where *F* and *G* are the finite element outputs and the predicted values by GP model, respectively; $\bar{F}$ and $\bar{G}$ are the average values of FE and GP data points, respectively; and n is the number of data points.

For the GP models, the correlation of coefficients (R^2^) and Mean Relative Error (*MRE*) are herein calculated for the Training and Validation, and the Test, subsets. Table 5 presents the calculated R^2^ and *MRE* for the proposed formulae. As per this table, it is seen that the R^2^ values are higher than 97 percent for the Train and Validation and over 96 percent for the Test data. Since the Test data had not been seen by the GP algorithm, and because the R^2^ and *MRE* values of the Train and Validation set are very close to the Test data, the models are able to predict the natural frequencies accurately and they do not overfit in the training process.

**Figure 8:**
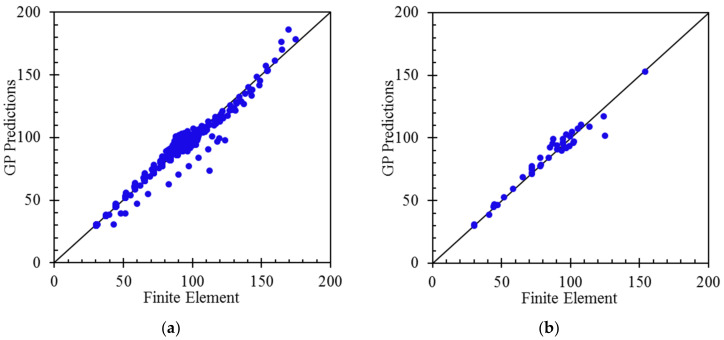

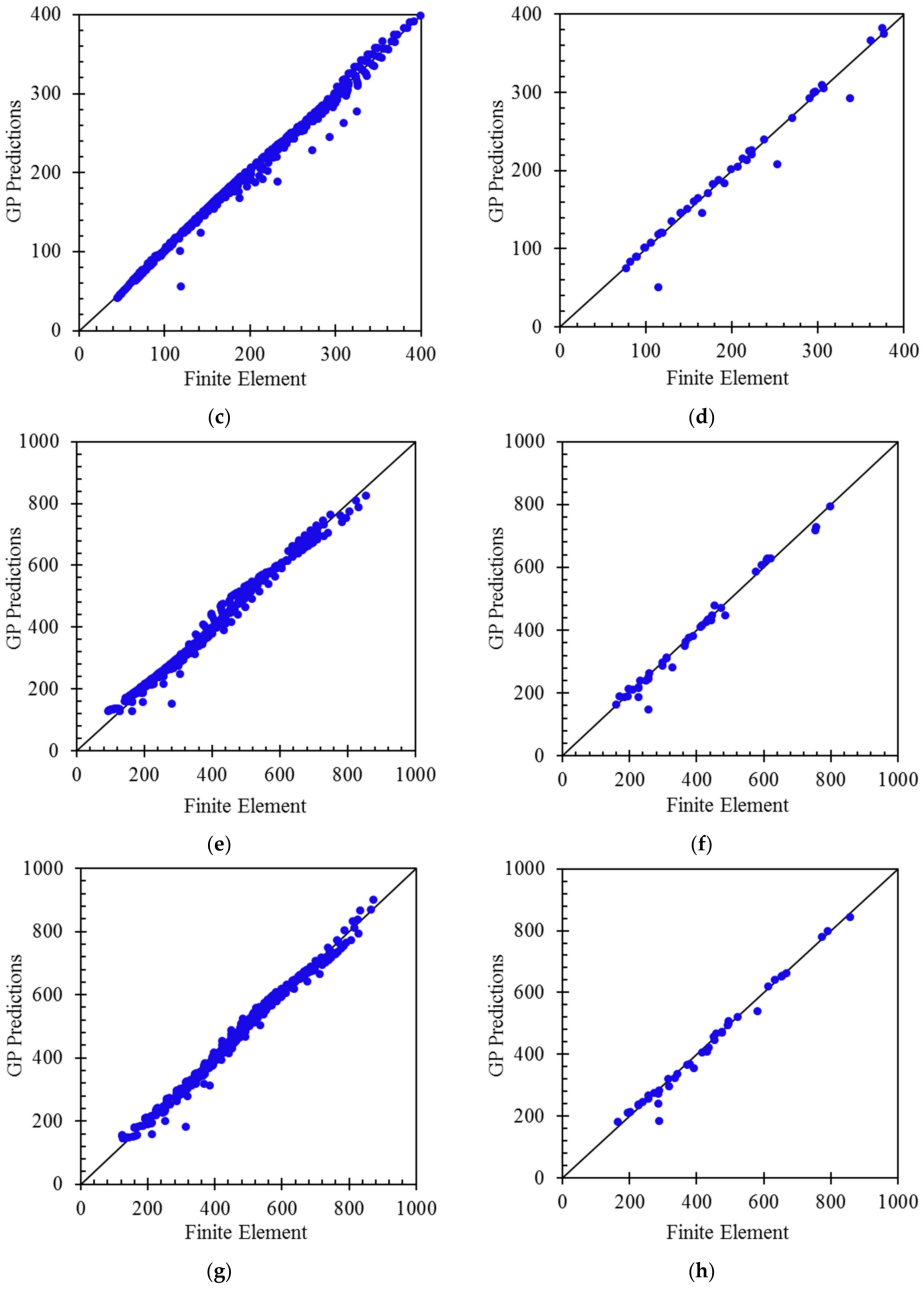
Comparison of the FE outputs with the predictions GP models for the natural frequencies: NF1 train and validation (**a**) and test (**b**) data; NF2 train and validation (**c**) and test (**d**) data; NF3 train and validation (**e**) and test (**f**) data; and NF4 train and validation (**g**) and test (**h**) data.

To have a better understanding of the models’ performance, Figure 8 presents the scatter diagrams of the GP models’ predictions versus the finite element data. According to these figures, a high correlation can be observed, and the data points are very close to the line of best fit.

For the external validation of the proposed formulas, it is required that at least the slope of one of the regression lines in Figure 5 (k and k’), which pass through the origin (0,0), be in the following range [56]:

(14) $$0.85<k,\;k^{\prime }<1.15$$

wherein

(15) $$k=\frac{1}{F^{2}}\sum F\times G$$

(16) $$k\prime =\frac{1}{G^{2}}\sum F\times G$$

where *F* and *G* are the finite element models’ outputs and the predicted values by GP formulas, respectively. The calculated values for the validation criteria are presented in Table 6.

### 5.3. Parametric Study and Sensitivity Analysis

**Figure 9:**
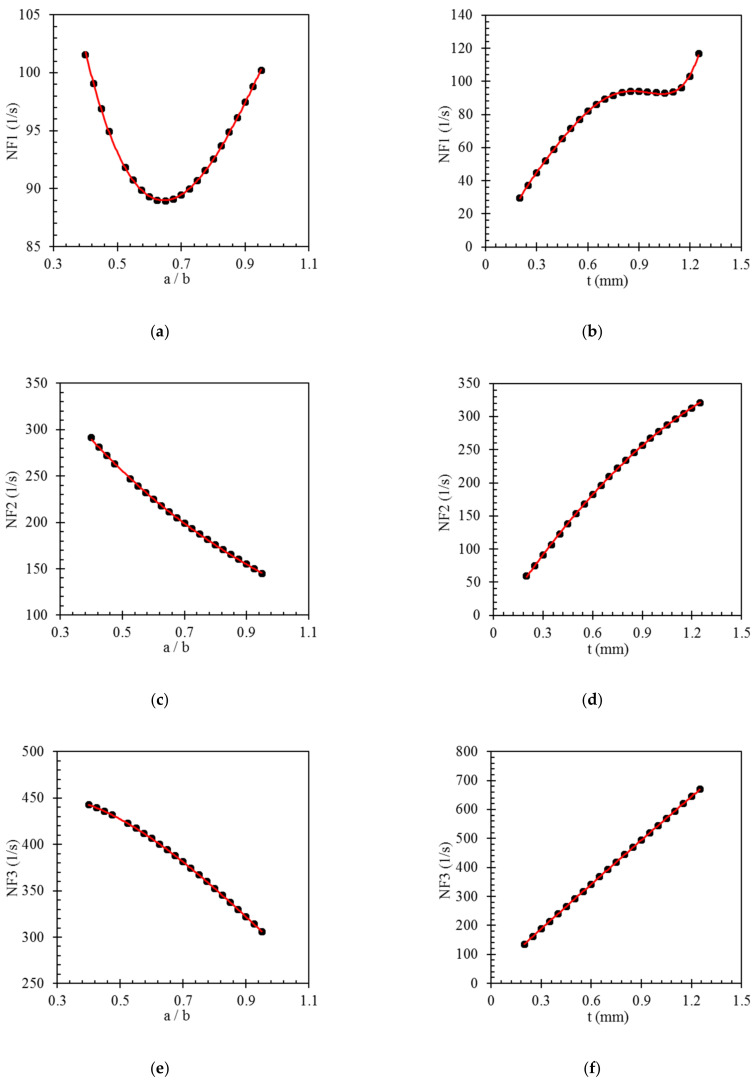

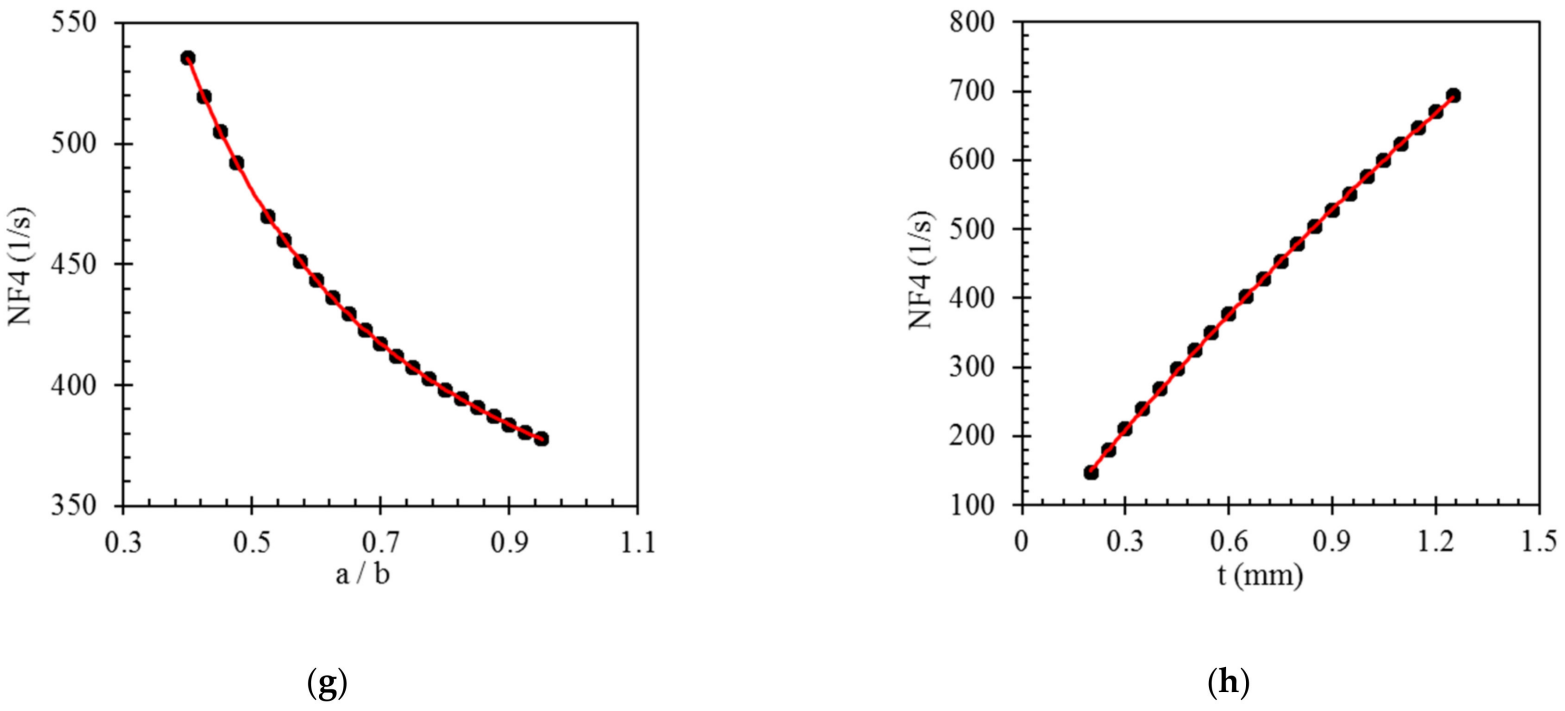
Results of the parametric studies on the inputs and output of the GP models. (**a**) the effect of *a/b* variation on NF1 (**b**) the effect of *t* variation on NF1 (**c**) the effect of *a/b* variation on NF2 (**d**) the effect of *t* variation on NF2 (**e**) the effect of *a/b* variation on NF3 (**f**) the effect of *t* variation on NF3 (**g**) the effect of *a/b* variation on NF4 (**h**) the effect of *t* variation on NF4.

Since the GP models are generally developed based on the best fit of the mathematical expressions on the data and not based on the physics of the problem, in order to present a clearer illustration of the relationships between the input parameters and the outputs, parametric studies are conducted on the four GP models. With regards to Figure 9, increasing the thickness, *t*, of the bistable composite laminate raises the natural frequencies. By considering Equations (6)–(8), increasing the thickness of the laminate can enhance the natural frequencies. However, increasing *a/b* leads to a reduction of the last three natural frequencies. In the case of the first natural frequency, rising *a/b* results in a rapid decrease of this natural frequency down to the critical length-to-width ratio. After that, increasing a/b causes a growth in this parameter, which forms a parabola. It should be mentioned that bistable laminates do not have two stable shapes for ratios that are equal to or less than the critical ratio, and the natural frequency in this point is zero [20,35].

In addition to the parametric studies that shed more light on the trend of the inputs and the output parameters with relevant physical interpretations, to find the level of contribution of the inputs (i.e., *a*/*b* and *t*), the percentage of the sensitivity of each model is calculated using the following formula [57]:

(17) $$D_{i}=NF_{max}\left(x_{i}\right)-NF_{min}\left(x_{i}\right)$$

(18) $$S_{i}=\frac{D_{i}}{\sum D}\left(\%\right)$$

wherein *NF_max_*(*x_i_*) and *NF_min_*(*x_i_*) are the maximum and minimum output values when the *i*th parameter is substituted in the GP formulas, while the average value of the other parameter is used. The results of the sensitivity analysis are presented in Table 7. According to the table, the natural frequencies of bistable laminates are more sensitive to the thickness rather than the length ratio. This is an advantage that makes it possible to design bistable structures with a desired vibration response by setting the thickness. It is seen that the contributions of the parameters in NF1 and NF4 are approximately similar, wherein the sensitivity of these two models to *a/b* is about one-fourth of *t*, which indicates the significantly higher contribution of the thickness in these modes. Nevertheless, different results can be seen for the sensitivity analysis of the second and third natural frequencies. Although these natural frequencies are more sensitive to the *a/b* compared to NF1 and NF4, thickness still plays a more vital role.

## 6. Conclusions

In the present research, the free vibration behaviour of the bistable laminates was investigated through the experiments and finite element analysis. Using the data acquired from the FE models, four new formulas were proposed using Multi-Objective Genetic Programming (MOGP) to predict the first four natural frequencies of bistable laminates just by considering their dimensions, leading to the tackling of the challenge to estimate these variables by means of closed-form nonlinear analytical expressions. The GP modelling started with splitting data to Training and Validation, and Test, data sets. The models were generated on the Training data and validated in each step of the population generation. Once the most efficient models (the highest correlation of coefficients and lowest error) were obtained, their accuracy was examined for predicting the natural frequencies of the Test dataset. After the justification of the GP mathematical formulas by FEM and the experiment’s test results, the ability of the GP formulas in predicting the desired parameters with high accuracy is notable. By analyzing the sensitivity of natural frequencies to input parameters, it can be seen that raising the thickness increases the natural frequencies, while growth of length sides’ ratio leads to general dwindling of the natural frequencies. As future work, this approach can be employed to obtain analytical equations for different responses of multistable or other complex structures.

## Figures and Tables

**Table 1:** The properties of (0/90) laminate [41].

$E_{11}\left[GPa\right]$	$E_{22}\;\left[GPa\right]$	$G_{12}\left[GPa\right]$	$\nu_{12}$	$\alpha_{11}\left[1/℃\right]$	$\alpha_{22\;}\left[1/℃\right]$	$t_{ply}\left[mm\right]$	$\Delta T\left[℃\right]$
147	10.71	6.98	0.3	$5.03\times 10^{-7}$	$2.65\times 10^{-5}$	0.225	160

**Table 2:** The optimum parameters of the MOGP models of this study.

Parameter	Setting
Functions	+, −, ×, /, exp, Ln, power, add3, mult3 ^1^
Population size	10,000
Number of generations	15,000
Maximum number of genes	2
Maximum tree depth	2
Training set	0.75
Validation set	0.15
Crossover events	0.85
High-level crossover	0.20
Low-level crossover	0.80
Sub-tree mutation	0.90

^1^ add3(a,b,c) = a + b + c, mult3(a,b,c) = a × b × c.

**Table 3:** The first four natural frequencies obtained by the FEA.

Natural Frequency (Hz)	1st	2nd	3rd	4th
FEA	33.81	63.91	130.22	143.19

**Table 4:** The predicted formulas for the first four natural frequencies (NF) by MOGP algorithm.

NF	Formula
NF1	$187.5x_{2}{}^{x_{2}{}^{x_{2}{}^{3.469}}}-3510x_{1}{}^{2}x_{2}{}^{1.351}\times {\left(1.911x_{1}{}^{2}\right)}^{x_{2}{}^{x_{2}}}\times exp\left(x_{2}-5.51x_{1}\right)-3.302$
NF2	$516.8-503.7\times {\left(0.5091x_{1}{}^{2.707}x_{2}\right)}^{0.5487x_{1}{}^{x_{1}}\times x_{2}{}^{x_{1}+1}}$
NF3	$704.2x_{2}{}^{\left(1.45\times 0.5467^{x_{1}}\right)}\times 1.469^{-1.809x_{1}{}^{2}}+27.13$
NF4	$450.6x_{2}\times {\left(x_{1}x_{2}{}^{2}\right)}^{-0.2237x_{1}}\times 1.039^{2x_{2}}\times 4.331^{{\left(0.0413\right)}^{x_{1}}}-40.43$

*^x^*_1_^= *a*/*b*, *x*^_2_^= *t*^

**Table 5:** The statistical indices for the Training and Validation, and the Test, subsets in the GP formulas.

Model	Train and Validation (75%, 15% Data)		Test (10% Data)	
	R^2^ (%)	*MRE* (%)	R^2^ (%)	*MRE* (%)
NF1	97.31	2.23	96.79	2.86
NF2	99.07	1.82	97.12	2.10
NF3	98.80	1.65	98.24	2.04
NF4	98.95	1.41	98.42	1.89

**Table 6:** The external validation results for the GP formulas.

Model	*k*	*k′*
NF1	0.993	1.002
NF2	0.997	1.002
NF3	0.999	0.998
NF4	1.000	0.999

**Table 7:** Results of the sensitivity analysis (*S_i_*) of the GP formulas.

Model	*S(a/b)%*	*S(t)%*
NF1	25.7	74.3
NF2	41.6	58.4
NF3	45.1	54.9
NF4	22.8	77.2

## Data Availability

The data supporting the findings of this study are available within the paper. Any other relevant data are also available upon reasonable request from the corresponding author.

## References

[1] Wu C., Viquerat A. (2018). Computational and experimental study on dynamic instability of extended bistable carbon/epoxy booms subjected to bending. Compos. Struct..

[2] Daynes S., Weaver P.M., Potter K.D. (2009). Aeroelastic study of bistable composite airfoils. J. Aircr..

[3] Nakhla S., Elruby A.Y. (2021). Applied Finite Element Procedure for Morphing Wing Design. Appl. Compos. Mater..

[4] Haldar A., Jansen E., Hofmeister B., Bruns M., Rolfes R. (2020). Analysis of novel morphing trailing edge flap actuated by multistable laminates. AIAA J..

[5] Li Y., Zhou S., Yang Z., Guo T., Mei X. (2019). High-performance low-frequency bistable vibration energy harvesting plate with tip mass blocks. Energy.

[6] Jiang G., Dong T., Guo Z. (2021). Nonlinear dynamics of an unsymmetric cross-ply square composite laminated plate for vibration energy harvesting. Symmetry.

[7] Fang S., Zhou S., Yurchenko D., Yang T., Liao W.-H. (2022). Multistability phenomenon in signal processing, energy harvesting, composite structures, and metamaterials: A review. Mech. Syst. Signal Process..

[8] Shore J., Viquerat A., Richardson G., Aglietti G. (2022). The natural frequency of drum-deployed thin-walled open tubular booms. Thin-Walled Struct..

[9] Zhang Z., Li Y., Yu X., Li X., Wu H., Wu H., Jiang S., Chai G. (2019). Bistable morphing composite structures: A review. Thin-walled Struct..

[10] Zhang Z., Yu X., Wu H., Sun M., Li X., Wu H., Jiang S. (2020). Non-Uniform Curvature Model and Numerical Simulation for Anti-Symmetric Cylindrical Bistable Polymer Composite Shells. Polymers.

[11] Sun M., Zhou H., Liao C., Zhang Z., Zhang G., Jiang S., Zhang F. (2022). Stable Characteristics Optimization of Anti-Symmetric Cylindrical Shell with Laminated Carbon Fiber Composite. Materials.

[12] Anilkumar P.M., Haldar A., Scheffler S., Jansen E.L., Rao B.N., Rolfes R. (2022). Morphing of bistable variable stiffness composites using distributed MFC actuators. Compos. Struct..

[13] Hyer M.W. (1981). Some observations on the cured shape of thin unsymmetric laminates. J. Compos. Mater..

[14] Akira H., Hyer M.W. (1987). Non-linear temperature-curvature relationships for unsymmetric graphite-epoxy laminates. Int. J. Solids Struct..

[15] Noor A.K., Burton W.S. (1992). Computational models for high-temperature multilayered composite plates and shells. Appl. Mech. Rev..

[16] Dano M.-L., Hyer M.W. (1998). Thermally-induced deformation behavior of unsymmetric laminates. Int. J. Solids Struct..

[17] Ren L., Parvizi-Majidi A., Li Z. (2003). Cured shape of cross-ply composite thin shells. J. Compos. Mater..

[18] Mattioni F., Weaver P.M., Potter K.D., Friswell M.I. (2008). Analysis of thermally induced multistable composites. Int. J. Solids Struct..

[19] Betts D.N., Salo A.I.T., Bowen C.R., Kim H.A. (2010). Characterisation and modelling of the cured shapes of arbitrary layup bistable composite laminates. Compos. Struct..

[20] Saberi S., Abdollahi A., Inam F. (2021). Reliability analysis of bistable composite laminates. AIMS Mater. Sci..

[21] Saberi S., Abdollahi A., Friswell M.I. (2021). Probability Analysis of Bistable Composite Laminates using the Subset Simulation Method. Compos. Struct..

[22] Brampton C.J., Betts D.N., Bowen C.R., Kim H.A. (2013). Sensitivity of bistable laminates to uncertainties in material properties, geometry and environmental conditions. Compos. Struct..

[23] Chai H., Li Y., Zhang Z., Sun M., Wu H., Jiang S. (2020). Systematic analysis of bistable anti-symmetric composite cylindrical shells and variable stiffness composite structures in hygrothermal environment. Int. J. Adv. Manuf. Technol..

[24] Fazli M., Sadr M.H., Ghashochi-Bargh H. (2021). Analysis of Bi-stable Hexagonal Composite Laminate Under Thermal Load. Appl. Compos. Mater..

[25] Deshpande V., Myers O., Fadel G., Li S. (2021). Transient deformation and curvature evolution during the snap-through of a bistable laminate under asymmetric point load. Compos. Sci. Technol..

[26] Phatak S., Myers O.J., Li S., Fadel G. (2021). Defining relationships between geometry and behavior of bistable composite laminates. J. Compos. Mater..

[27] Nicassio F. (2021). Shape prediction of bistable plates based on Timoshenko and Ashwell theories. Compos. Struct..

[28] De Pietro G., Giunta G., Hui Y., Belouettar S., Hu H., Carrera E. (2021). A novel computational framework for the analysis of bistable composite beam structures. Compos. Struct..

[29] Kuang Z., Huang Q., Huang W., Yang J., Zahrouni H., Potier-Ferry M., Hu H. (2021). A computational framework for multi-stability analysis of laminated shells. J. Mech. Phys. Solids.

[30] Zhang Z., Pei K., Sun M., Wu H., Wu H., Jiang S., Zhang F. (2022). Tessellated multistable structures integrated with new transition elements and antisymmetric laminates. Thin-Walled Struct..

[31] Diaconu C.G., Weaver P.M., Arrieta A.F. (2009). Dynamic analysis of bi-stable composite plates. J. Sound Vib..

[32] Arrieta A.F., Neild S.A., Wagg D.J. (2011). On the cross-well dynamics of a bi-stable composite plate. J. Sound Vib..

[33] Wu C., Viquerat A. (2017). Natural frequency optimization of braided bistable carbon/epoxy tubes: Analysis of braid angles and stacking sequences. Compos. Struct..

[34] Wu Z., Li H., Friswell M.I. (2018). Advanced nonlinear dynamic modelling of bi-stable composite plates. Compos. Struct..

[35] Emam S.A. (2018). Snapthrough and free vibration of bistable composite laminates using a simplified Rayleigh-Ritz model. Compos. Struct..

[36] Wu M.Q., Zhang W., Niu Y. (2021). Experimental and numerical studies on nonlinear vibrations and dynamic snap-through phenomena of bistable asymmetric composite laminated shallow shell under center foundation excitation. Eur. J. Mech..

[37] Lee A.J., Inman D.J. (2019). Electromechanical modelling of a bistable plate with macro fiber composites under nonlinear vibrations. J. Sound Vib..

[38] Brunetti M., Mitura A., Romeo F., Warminski J. (2022). Nonlinear dynamics of bistable composite cantilever shells: An experimental and modelling study. J. Sound Vib..

[39] Liu T., Zhang W., Wu M.Q., Zheng Y., Zhang Y.F. (2021). Metastable nonlinear vibrations: Third chaos of bistable asymmetric composite laminated square shallow shell under foundation excitation. Compos. Struct..

[40] Dong T., Guo Z., Jiang G. (2021). Global and Local Dynamics of a Bistable Asymmetric Composite Laminated Shell. Symmetry.

[41] Saberi S., Ghayour M., Mirdamadi H.R., Ghamami M. (2021). Free vibration analysis and mode management of bistable composite laminates using deep learning. Arch. Appl. Mech..

[42] Castro S.G.P., Guimarães T.A.M., Rade D.A., Donadon M.V. (2016). Flutter of stiffened composite panels considering the stiffener’s base as a structural element. Compos. Struct..

[43] Castro S.G.P., Donadon M. (2017). V Assembly of semi-analytical models to address linear buckling and vibration of stiffened composite panels with debonding defect. Compos. Struct..

[44] Castro S.G.P., Zimmermann R., Arbelo M.A., Degenhardt R. (2013). Exploring the constancy of the global buckling load after a critical geometric imperfection level in thin-walled cylindrical shells for less conservative knock-down factors. Thin-Walled Struct..

[45] Castro S.G.P., Zimmermann R., Arbelo M.A., Khakimova R., Hilburger M.W., Degenhardt R. (2014). Geometric imperfections and lower-bound methods used to calculate knock-down factors for axially compressed composite cylindrical shells. Thin-Walled Struct..

[46] Koza J.R. (1994). Genetic programming as a means for programming computers by natural selection. Stat. Comput..

[47] Braik M. (2021). A hybrid multi-gene genetic programming with capuchin search algorithm for modeling a nonlinear challenge problem: Modeling industrial winding process, case study. Neural Process. Lett..

[48] Kusznir T., Smoczek J. (2022). Multi-Gene Genetic Programming-Based Identification of a Dynamic Prediction Model of an Overhead Traveling Crane. Sensors.

[49] Gao Y., Ye J., Yuan Z., Ling Z., Zhou Y., Lin Z., Dong J., Wang H., Peng H.-X. (2022). Optimization strategy for curing ultra-thick composite laminates based on multi-objective genetic algorithm. Compos. Commun..

[50] Rajabi Z., Eftekhari M., Ghorbani M., Ehteshamzadeh M., Beirami H. (2022). Prediction of the degree of steel corrosion damage in reinforced concrete using field-based data by multi-gene genetic programming approach. Soft Comput..

[51] Alavi A.H., Gandomi A.H. (2011). A robust data mining approach for formulation of geotechnical engineering systems. Eng. Comput. Int. J. Comput. Eng..

[52] Gandomi A.H., Alavi A.H. (2012). A new multi-gene genetic programming approach to non-linear system modeling. Part II: Geotechnical and earthquake engineering problems. Neural Comput. Appl..

[53] Searson D.P. (2015). GPTIPS 2: An open-source software platform for symbolic data mining. Handbook of Genetic Programming Applications.

[54] Frank I.E., Todeschini R. (1994). The Data Analysis Handbook.

[55] Smith G.N. (1986). Probability and Statistics in Civil Engineering.

[56] Golbraikh A., Tropsha A. (2002). Beware of q2!. J. Mol. Graph. Model..

[57] Sadat Hosseini A., Hajikarimi P., Gandomi M., Moghadas Nejad F., Gandomi A.H. (2021). Genetic programming to formulate viscoelastic behavior of modified asphalt binder. Constr. Build. Mater..

